# Not So Rare: Diseases Based on Mutant Proteins Controlling Endoplasmic Reticulum-Mitochondria Contact (MERC) Tethering

**DOI:** 10.1177/25152564241261228

**Published:** 2024-07-25

**Authors:** Tadashi Makio, Thomas Simmen

**Affiliations:** 1 Department of Cell Biology, Faculty of Medicine and Dentistry, University of Alberta, Edmonton, Alberta, Canada

**Keywords:** endoplasmic reticulum, mitochondria, rare disease, mutation

## Abstract

Mitochondria-endoplasmic reticulum contacts (MERCs), also called endoplasmic reticulum (ER)-mitochondria contact sites (ERMCS), are the membrane domains, where these two organelles exchange lipids, Ca^2+^ ions, and reactive oxygen species. This crosstalk is a major determinant of cell metabolism, since it allows the ER to control mitochondrial oxidative phosphorylation and the Krebs cycle, while conversely, it allows the mitochondria to provide sufficient ATP to control ER proteostasis. MERC metabolic signaling is under the control of tethers and a multitude of regulatory proteins. Many of these proteins have recently been discovered to give rise to rare diseases if their genes are mutated. Surprisingly, these diseases share important hallmarks and cause neurological defects, sometimes paired with, or replaced by skeletal muscle deficiency. Typical symptoms include developmental delay, intellectual disability, facial dysmorphism and ophthalmologic defects. Seizures, epilepsy, deafness, ataxia, or peripheral neuropathy can also occur upon mutation of a MERC protein. Given that most MERC tethers and regulatory proteins have secondary functions, some MERC protein-based diseases do not fit into this categorization. Typically, however, the proteins affected in those diseases have dominant functions unrelated to their roles in MERCs tethering or their regulation. We are discussing avenues to pharmacologically target genetic diseases leading to MERC defects, based on our novel insight that MERC defects lead to common characteristics in rare diseases. These shared characteristics of MERCs disorders raise the hope that they may allow for similar treatment options.

## Introduction

### The Discovery of Mitochondria-Endoplasmic Reticulum Contact Sites (MERCs) as Mitochondria-Associated Membranes (MAMs)

The original discovery by the Bernhard lab of mitochondria and endoplasmic reticulum (ER) contact sites (MERCs) showed over 70 years ago that these interorganellar contacts respond acutely to distinct metabolic and feeding states (Bernhard et al., 1952). We have reviewed the history of MERCs research recently (Herrera-Cruz and Simmen, 2017). This and other early findings remained obscure for a long time but suggested that MERCs intricately adapt to a variety of conditions, and that their putative functions could be compromised in a disease setting. Close to 40 years passed until MERC functions found solid experimental evidence with the discovery of MAMs as a lipid transfer platform (Vance, 1990) and a site of Ca^2+^ flux (Rizzuto et al., 1998). Both findings were key to the acceptance of MERCs as a true functional entity. The discovery of inflammasome formation and reactive oxygen species (ROS) nanodomains at MERCs added their connection to oxidative stress signaling (Zhou et al., 2011; Booth et al., 2016). From these seminal studies, many additional functions have emerged, which we will summarize in the following sections.

### MERCs as Synthesis and Transfer Hubs for Lipids

**Figure 1.**
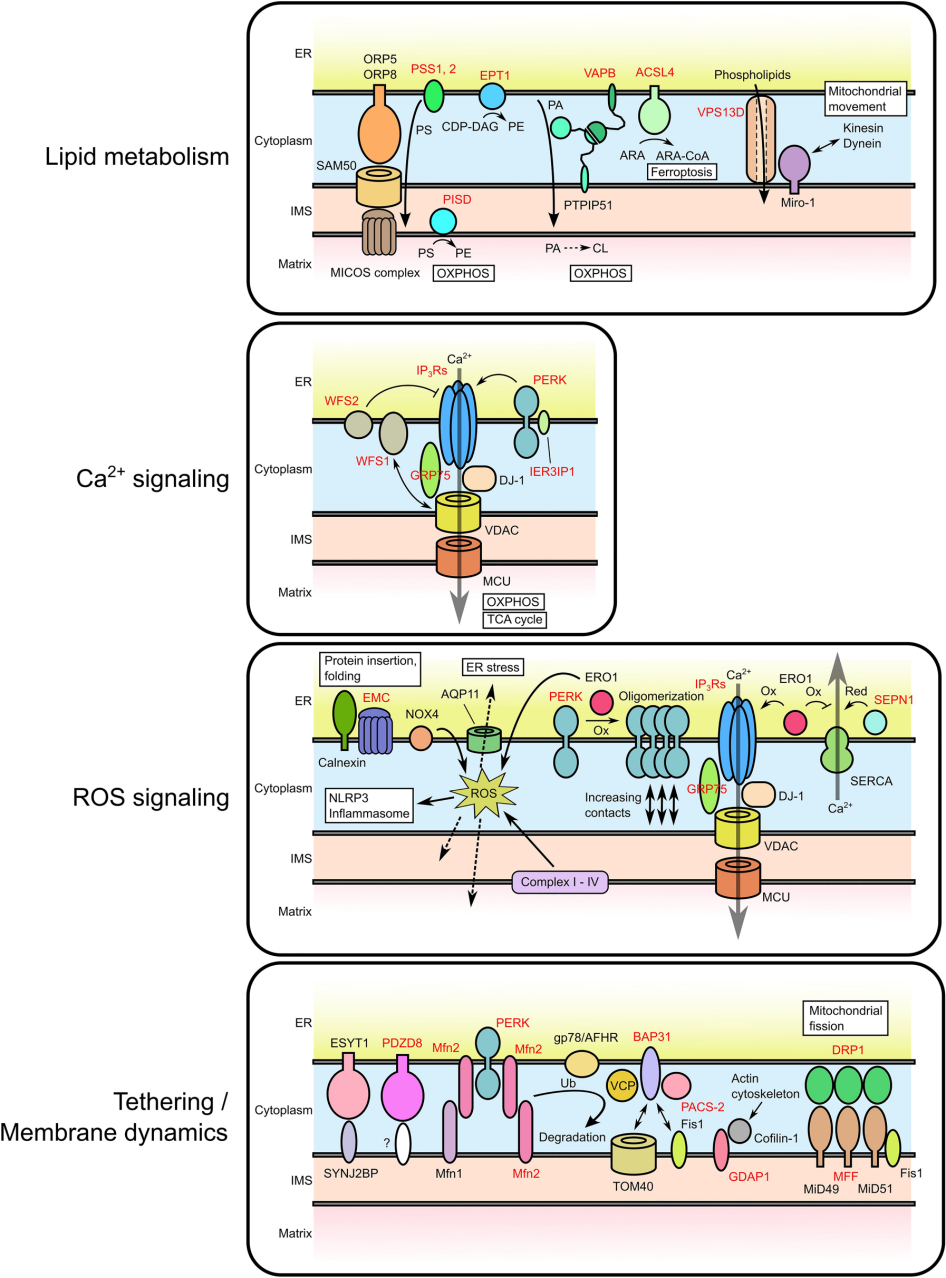
Functioning of MERC tethers and regulatory proteins associated with rare diseases. The schematics of the most important MERCs proteins and their functions are presented. For detail, please refer to the corresponding main text. The genes whose mutations have been known to cause rare diseases are shown in red. The proteins are categorized by their functions. (Top) The proteins involved in phospholipid transfer/metabolism at MERCs. (Second) The proteins involved in Ca^2+^ signaling and its regulation. (Third) The proteins involved in ROS signaling. (Bottom) The proteins involved in MERC tethering and mitochondrial membrane dynamics.

*Note*. MERCs: Mitochondria-endoplasmic reticulum contact sites; ROS = reactive oxygen species.

The first function detected on MERCs was lipid metabolism (Figure 1, top panel) through the presence of enzymes synthesizing phosphatidylserine (PS; PS synthases 1 and 2 [PSS1, PSS2]), and phosphatidylcholine (PC; phosphatidylethanolamine methyltransferase [PEMT]) (Vance, 1991). The expression of these enzymes is highest in liver tissue, where they are frequently used as MERCs/MAMs marker proteins (Stone and Vance, 2000). A MAM marker that is more widely expressed is acyl-CoA synthetase long-chain family member 4 (ACSL4), also known as fatty acid-CoA ligase 4 (FACL4), which ligates fatty acids to coenzyme A (CoA) and controls polyunsaturated fatty acid (PUFA) synthesis (Lewin et al., 2001, 2002). These enzymes will be discussed in detail regarding their involvement in MERC rare diseases.

Following the identification as a lipid synthesis hub, MAMs were identified as the ER membrane, where PS is transferred to mitochondria, followed by its transformation into phosphatidylethanolamine (PE) on the inner mitochondrial membrane (Vance, 2003). PS-PE conversion is catalyzed by mitochondrial phosphatidyl decarboxylase (PISD), in parallel to ER-localized ethanolamine phosphotransferase 1 (EPT1) (Vance, 2015). While vesicular or direct cytosolic transport could not be detected for this lipid flux (Voelker, 1989), it requires ATP in mammalian liver cells, suggesting ongoing mitochondrial oxidative phosphorylation (OXPHOS) is required for this MERC function (Tatsuta et al., 2014). Recent findings indicate that MERC bulk lipid flux in general is mediated by the Vps13D lipid channel (Guillen-Samander et al., 2021). Smaller scale lipid exchange occurs through the pairing of ER oxysterol binding protein-related proteins 5 and 8 (ORP5, ORP8) (Guyard et al., 2022) with the mitochondrial contact sites and cristae junction organizing system (MICOS) (Monteiro-Cardoso et al., 2022). Given the importance of lipid synthesis and metabolism for liver tissue (Nguyen et al., 2008), the knockout of MERC-associated PSS1 and PSS2 was initially found to compromise liver functions (Bergo et al., 2002; Arikketh et al., 2008).

Another lipid transiting from the ER to mitochondria is phosphatidic acid (PA), which subsequently converts into mitochondrial cardiolipin (CL) with the help of a cascade of enzymes that culminates in CL synthase (Yeo et al., 2021). This cone-shaped lipid is critical for the classic curvature of mitochondrial cristae (Beltran-Heredia et al., 2019) and essential for the supercomplex assembly mediating OXPHOS (Zhang et al., 2002; Pfeiffer et al., 2003). The three-dimensional structure of cristae, as dictated by their enrichment of cone-shaped lipids, is thus a key requirement for the efficiency of mitochondrial respiration and cellular bioenergetics overall (Cogliati et al., 2013). This function is particularly evident in tissues with high energy demand (Finsterer, 2019). To a lesser extent, cone-shaped PE also promotes the mitochondrial OXPHOS capacity (Boyd et al., 2017; Funai et al., 2020). Since CL and PE are so important for mitochondrial ATP production, their synthesis is expected to predominantly impact mitochondrial functions, and thus predict weak MERC dependence of these pathologies. Indeed, defective CL synthesis in Barth syndrome (Mendelian Inheritance in Man, MIM#302060) is considered a mitochondrial disease (Finsterer, 2019). The rare X-linked pathology is characterized by cardiac and skeletal myopathy, as well as growth retardation in early life (Clarke et al., 2013). Unlike typical MERC-associated diseases (see later), Barth syndrome only affects the brain to a minor extent, compromising sensory perception, and leading to fatigue and minor cognitive defects (Olivar-Villanueva et al., 2021). In contrast, the importance of PE for the brain is more pronounced, maybe because it is not as essential for mitochondria and also, since PE is particularly abundant in this tissue (Vance and Tasseva, 2013). Within the brain, the significance of EPT1-associated PE synthesis is particularly obvious within motor neurons, where EPT1 mutations lead to hereditary spastic paraplegia (MIM#618768; Ahmed et al., 2017). However, similar to CL, its promotion of mitochondrial OXPHOS is also important for the functioning of skeletal muscle (Heden et al., 2019), and brown adipose tissue (Johnson et al., 2023). Together, MERC lipid synthesis and flux emerge as a key factor for mitochondrial ATP production. This function is tightly associated with the role of lipids like CL and PE for membrane curvature, a key feature needed for mitochondrial OXPHOS on their cristae. Thus, the functional readout of MERC-associated lipid metabolism is predominantly mitochondrial and is critical for liver, brain, and muscle tissue, leading to broad-spectrum disease if the genes encoding these proteins are mutated.

### MERCs as a Ca^2+^ Signaling Center

The metabolic significance of MERCs is perhaps even more obvious from their role as a center of intracellular Ca^2+^ signaling (Figure 1, second panel). On MERCs, ER inositol 1,4,5-trisphosphate receptors (IP_3_Rs) interact with mitochondrial voltage-dependent ion channels (VDACs), assisted by the mitochondrial chaperone GRP75 (Szabadkai et al., 2006). IP_3_Rs can also interact with mitochondrial TOM70 (Filadi et al., 2018a). Therefore, the formation of these multimeric protein complexes is essential not only for the actively triggered Ca^2+^ transfer from the ER to mitochondria, but also for MERC tethering, suggesting the two functions overlap. Potentially, this is further promoted by mitochondrial arrest in locations with high [Ca^2+^] (Saotome et al., 2008; Wang and Schwarz, 2009). More importantly for the topic of this review, IP_3_R-originating Ca^2+^ flux controls the activity of mitochondrial Krebs cycle enzymes and, thus, mitochondrial respiration and ATP production (Cardenas et al., 2010).

To our knowledge, the relative importance of MERC lipid and Ca^2+^ flux for mitochondrial metabolism has never been quantified and such an endeavor might prove challenging. As we will discuss further below and in contrast to some of the lipid metabolic enzymes, mutations in proteins mediating or regulating MERC Ca^2+^ flux do not fully compromise mitochondria, but rather reduce their relative contribution to cellular bioenergetics and increase compensatory glycolysis, thus providing a hallmark change upon MERC interference (Tarasov et al., 2012). Another emerging theme of this review is that MERCs are critical for the central and peripheral nervous systems (CNS/PNS). This is also highlighted by the large number of MERC Ca^2+^-handling proteins associated with neurodegeneration (Krols et al., 2016). Prominent members of this group are the presenilins, genetic loci for Alzheimer's disease (AD) (Area-Gomez et al., 2009), which control mitochondrial Ca^2+^ uptake at the synapse, where these organelles provide much of the energy needed for neurotransmitter release (Walters and Usachev, 2023). Accordingly, mutant presenilin-2 decreases the ER Ca^2+^ content by reducing the activity of sarco-endoplasmic reticulum Ca^2+^ ATPase (SERCA; Green et al., 2008), which increases Ca^2+^ flux from the ER to mitochondria (Zampese et al., 2011). Through this Ca^2+^ imbalance, MERCs increase in AD patient and mouse model tissue (Hedskog et al., 2013). However, presenilin mutant proteins also increase PE synthesis and promote MERC formation, again suggesting a tight interweaving of these two functions (Area-Gomez et al., 2012). Given that mutations within the presenilin genes are not considered rare, we will not further discuss them in this review. Similarly, MERC Ca^2+^ flux, dependent on IP_3_Rs, VDACs and the mitochondrial Ca^2+^ uniporter (MCU) plays an important role in many tissues and diseases, for instance diabetes (Rieusset, 2017). In terms of mutations leading to disease, gain-of-function mutations of IP_3_Rs that result in spinocerebellar ataxia are the basis of rare diseases, discussed in detail later (Casey et al., 2017). Therefore, MERC Ca^2+^ flux controls the qualitative contribution of mitochondria to bioenergetics, which leads to a less mitochondria-centric disease array with implications for a wide variety of tissues such as the pancreas, but most critically for the CNS.

### MERCs as a Target and Source of Oxidative Stress

The latest addition to the canon of MERC functions was discovered in 2011 through the localization of the NLRP3 inflammasome to MERCs (Zhou et al., 2011) in the presence of oxidative stress signaling (Figure 1, third panel). This key regulator of inflammation depends on the production of ROS within mitochondria and the ER, which triggers association of thioredoxin interacting protein (TXNIP) with the inflammasome (Zhou et al., 2010). While normally acting as an inhibitor of the antioxidant thioredoxins, TXNIP association with the inflammasome further potentiates ROS levels especially if associated with mitochondria (Saxena et al., 2010). This function predicts that the interaction between TXNIP and the NLRP3 inflammasome depends on the formation of MERC redox nanodomains, whose ROS content oxidizes IP_3_Rs, thus activating MERC Ca^2+^ signaling (Booth et al., 2016, 2021). Oxidation of cysteines is initiated by their sulfenylation, followed by sulfinylation of responsive cysteine residues, both of which are reversible through the activity of sulforedoxin. Therefore, cysteine oxidation acts as a posttranslational modification not unlike phosphorylation (Paulsen and Carroll, 2013). Upon further oxidation, cysteines can undergo sulfonylation, which is irreversible (Yang et al., 2016). Each of these modifications changes the function of substrates and may achieve their activation through oligomerization (Bassot et al., 2021).

MERCs are a hotspot for such modifications, since ER and mitochondria release ROS through pores like aquaporin-11 (AQP11) (Bestetti et al., 2020). This ROS signaling allows MERCs to respond to stress. This can occur, for instance, from the accumulation of unfolded proteins within the ER (Bassot et al., 2023), a condition long known to increase MERCs lipid and Ca^2+^ flux through the tightening of the contacts (Csordas et al., 2006; Bravo et al., 2011). While mitochondrial ROS are thought to derive from OXPHOS imbalance (Murphy, 2009), the ER ROS sources with MERC-modulating potential have been identified as NOX4 that seems to promote baseline activity of MERCs (Gutierrez et al., 2020) and the ER oxidoreductase ERO1L that oxidizes the eIF2α kinase 3/protein kinase R like ER kinase (PERK) (Bassot et al., 2023). Thus, PERK acts as a stress-induced tether at MERCs (Verfaillie et al., 2012) and as an activator of MERC Ca^2+^/lipid flux that switches cell metabolism from glycolysis to OXPHOS (Bassot et al., 2023). Although the cytosolic MERC interface might directly interact with NOX enzymes (Dikalov, 2011), we currently do not know a specific cytosolic MERC redox enzyme that could modulate mitochondria or ER redox release. Such a postulated mechanism is under the control of cytosolic glutathione, thioredoxins, and peroxiredoxins (Jezek et al., 2020).

The link between ROS signaling and MERC lipid metabolism is currently poorly understood. Upon disruption of PS or PC synthesis, ROS levels go up, potentially through the disruption of efficient OXPHOS, and these ROS appear to predominantly affect glial cells, at least in the fly model, leading to a neurodegenerative phenotype (Park et al., 2021). Conversely, several MERC lipid and cholesterol metabolic enzymes (e.g., ACAT1) are targets of ROS-mediated oxidation (Akter et al., 2018). For instance, acyl-coA:acyltransferase 2 (ACAT2) undergoes sulfenylation, which activates and stabilizes this producer of cholesteryl esters (Wang et al., 2017). In contrast, the oxidation of MERC-enriched diacyglycerol acyltransferase 2 (DGAT2) (Stone et al., 2009) inactivates it (Jung et al., 2017), indicating that MERC ROS levels profoundly affect lipid metabolism.

Another topic in this context is the MERC-specific peroxidation of PUFAs during ferroptosis (Dixon et al., 2012; Stockwell, 2022). The MERC marker ACSL4/FACL4 promotes the production of PUFAs through the incorporation of arachidonic acid into phospholipids (Kang et al., 1997). It is therefore not surprising that ACSL4 is a known ferroptosis regulator (Doll et al., 2017). Once peroxidized, lipids can revert to their reduced form through the activity of GPx4 protein, a selenoperoxidase found in the cytoplasm, within mitochondria and the nucleus (Xie et al., 2023). Lipid peroxidation reveals an association of MERC oxidative signaling with mitochondria homeostasis, since mitochondrial ROS production creates MERC redox nanodomains (Booth et al., 2016). Accordingly, the interference with MERC formation also blocks ferroptosis (Zhang et al., 2023). Aside from the ERO1L interactor SELENON that we discuss later, given the largely unknown identity of MERC redox regulators, no rare disease is currently known that is tied to defective MERC redox signaling.

### MERCs Mediate Stress-Dependent Metabolic Signaling

The intimate link between mitochondrial and ER redox conditions explains why ER stress is induced upon inhibiting mitochondrial Krebs cycle activity (Kuznetsov et al., 2022). A mechanistic MERC-associated basis for diseases resulting from direct mutations of ER stress and mitochondrial Krebs cycle enzymes might therefore prove difficult to untangle from their other functions. Nevertheless, the now established link between ER stress signaling and MERC metabolic signaling (Simmen and Herrera-Cruz, 2018) explains why gene mutations within *PERK* are particularly relevant to this review. PERK is best known as a moderator of ER protein synthesis through eIF2α phosphorylation during the induction of the unfolded protein response (UPR) (Ron and Walter, 2007), but also as a MERC-localized interactor with the tethers mitofusin-2 (Munoz et al., 2013) and extended synaptotagmin-1 (E-Syt1) to promote mitochondrial respiration (Sassano et al., 2023). Both E-Syt1 (Chang et al., 2013) and PERK (van Vliet et al., 2017) also localize to ER-plasma membrane junctions, thus implicating them in store-operated Ca^2+^ entry (SOCE) (Chen et al., 2019; Kang et al., 2019). Mutations in *PERK* cause Wolcott-Rallison syndrome (MIM#226980) that leads to neonatal diabetes but also epileptic seizures (de Wit et al., 2006; Julier and Nicolino, 2010). Whether this disease spectrum depends more on the role of PERK in the integrated stress response and, thus, the prevention of further ER stress (Pakos-Zebrucka et al., 2016) or on its role in MERC tethering to maintain mitochondria metabolism (van Vliet and Agostinis, 2016) remains unclear. Regardless, Wolcott-Rallison syndrome is reminiscent of Wolfram syndrome (MIM#222300), which leads to diabetes mellitus, optic nerve atrophy, central diabetes insipidus, sensorineural deafness, urinary tract problems, and progressive neurologic difficulties (Urano, 2016). Wolfram syndrome is caused by mutations in two genes, *WFS1* and *WFS2* (Pallotta et al., 2019). Both proteins encoded by these genes are of interest for this review. WFS1 is a glycosylated transmembrane protein that localizes to the ER, where it not only promotes mitochondrial membrane contact formation but is also a major determinant of the Ca^2+^-signaling functions at MERCs (Angebault et al., 2018). Due to its interaction with VDAC and the important role of ER-mitochondria Ca^2+^ flux for cell metabolism, it comes as no surprise that WFS1 maintains mitochondrial ATP production (Zatyka et al., 2023). The *WFS2* gene product, CDGSH iron–sulfur domain-containing protein 2 (CISD2), is also an integral ER membrane protein that interacts with and inhibits IP_3_Rs, as well as autophagy (Chang et al., 2010). Moreover, CISD2 localizes to MERCs and prevents ER stress, possibly through its function as a preserver of mitochondrial bioenergetics (Wiley et al., 2013).

Another connection between ER stress and MERC metabolic signaling is revealed in the syndrome of microcephaly, epilepsy, and permanent neonatal diabetes (MEDS, MIM#614231), which is based on mutations within the immediate early response 3 interacting protein 1 (*IER3IP1)* gene (Poulton et al., 2011; Yang et al., 2022). IER3IP1 is an ER transmembrane protein (Yiu et al., 2004) that regulates ER protein export towards the Golgi complex and prevents ER stress (Esk et al., 2020). In this case, and for all proteins discussed up to here, the connection of the diseases to MERC dysfunction is currently unclear. However, as we will outline later, some aspects of these pathologies are very reminiscent of the syndrome resulting from mutations in the gene encoding the cytosolic phosphofurin acidic cluster sorting protein 2 (PACS-2) (Simmen et al., 2005).

### MERCs and Mitochondrial Dynamics

Mitochondrial dynamics control the fission and fusion of mitochondria, which controls the levels of mitochondrial stress and distribution of mitochondria within the cell (Chan, 2020). This function is tightly linked to MERC tethering (Figure 1, bottom panel). Mitochondrial fusion is mediated by the pairing of the dynamin superfamily GTPases mitofusin 1 and 2 (Mfn1/Mfn2) on neighboring outer mitochondrial membranes (OMM) (Koshiba et al., 2004). Mfn2 and its mutants will be discussed in detail later, since ER- and mitochondria-specific splice variants act as key MERC tethers (Naon et al., 2023). In parallel, the inner mitochondrial membrane (IMM) GTPase Opa1 mediates the second step of fusion (Song et al., 2007). The fusion and elongation of mitochondria is tightly connected to MERCs, as evidenced by PERK activation, which induces stress-dependent mitochondria elongation (Lebeau et al., 2018), accompanied by the accumulation of the CL precursor PA on the OMM (Perea et al., 2023).

Counteracting fusion, mitochondrial fission aims to increase the number of mitochondria. This happens, for instance, during excessive stress that can trigger the phosphorylation (Cribbs and Strack, 2007) and oxidation (Cho et al., 2009) of the GTPase dynamin-related protein 1 (Drp1). Subsequently, Drp1 associates with the OMM proteins Fis1, Mff, MiD49, and MiD51 (Loson et al., 2013). Importantly, the fission machinery is assembled on MERCs (Friedman et al., 2011) with the help of these receptor proteins (Ji et al., 2017). Mutations within the genes encoding the MERC-associated fission machinery share many characteristics with MERC dysfunction-based pathologies, as discussed below.

MERC formation indirectly depends on mitochondria motility along the cytoskeleton, which could be an initial determinant of ER-mitochondria contact formation (Yi et al., 2004). A key factor for mitochondria movement is the small OMM Rho protein 1 (Miro-1), a GTPase enriched in yeast and mammalian MERCs (Modi et al., 2019). Miro-1 is conserved from humans to yeast, where its counterpart Gem1 is a component of the yeast ERMES tethering complex (Kornmann et al., 2011). On the membrane contact site, Miro-1 senses high cytosolic [Ca^2+^] released from IP_3_Rs through its EF hand, which arrests mitochondria movement (Saotome et al., 2008; Wang and Schwarz, 2009). Thus, Miro-1 conveys Ca^2+^-sensitivity to mitochondria positioning (Fransson et al., 2006; Eberhardt et al., 2020). This is achieved through the interaction of Miro-1 with the kinesin and dynein motor proteins (Macaskill et al., 2009; Morlino et al., 2014). The Miro-1 activity is critically important for axonal transport of mitochondria to synapses, firstly shown in Drosophila (Guo et al., 2005). Similar to the presenilins, mutant Miro-1 is observed in Parkinson's disease (Grossmann et al., 2020).

To conclude the introduction, MERC-associated proteins are connected to a variety of diseases, ranging from neurological, muscle, to pancreatic defects. Given their frequent multifunctional aspects, many of these may depend on functions not related to MERCs. In the case of defective mitochondrial dynamics, as related to MERC assembly, such diseases are primarily associated with defects within the central and peripheral nervous system.

## Rare Diseases Based on MERC Tethers

As outlined above, MERC functions comprise exchange of lipids, Ca^2+^, and ROS that then impinge on metabolic functions of mitochondria to a varying extent. Changes in lipid homeostasis appear to act most directly, while Ca^2+^ signaling acts more subtly and the impact of interfering with MERC redox nanodomains is currently largely unexplored. To gain better assessment of the significance of MERC interference for disease, we will first focus on the physical tethering between the ER and mitochondria. We will further narrow the discussion and exclude proteins are either associated to common diseases (e.g., DJ-1, presenilins) or do not primarily control MERC formation or regulation (e.g., ACSL4, EPT1, PERK, WFS1, CISD2, or IER3IP1). When doing so, we detect highly similar pathologies.

MERC tethering is controlled by a set of proteins that bridge the gap between the two organelles. Typically, tethers are formed by different proteins that are found on the ER or mitochondria, respectively. For example, ER-localized IP_3_Rs can bridge the two organelles through interaction with mitochondrial VDAC and the chaperone GRP75 (Szabadkai et al., 2006). This function also depends on the mitochondrial protein DJ-1 (Ottolini et al., 2013; Liu et al., 2019), a cytosolic protein that interacts with glycolytic enzymes and prevents ROS formation as a chaperone, but relocates to mitochondrial upon its oxidation (Mencke et al., 2021). One of the best-characterized tethering pair is formed between ER-localized VAPB (VAMP-associated membrane protein B) that connects to mitochondrial protein tyrosine phosphatase interacting protein 51 (PTPIP51), also called regulator of microtubules dynamics 3 (RMDN3) (De Vos et al., 2012; Stoica et al., 2014). ER-localized proteins like gp78/AMFR connect to these and determine the extent of tethering (Wang et al., 2015). Another protein complex is the ARCosome that is formed when ER-localized BAP31 (also known as BCAP31) interacts with mitochondrial Fis1 (Iwasawa et al., 2011) and with the import complex component TOM40 (Namba, 2019). More recently, a protein complex between ER-localized E-Syt1 and mitochondrial SYNJ2BP has been identified (Janer et al., 2024). Given that E-Syt1 is recruited by PERK to MERCs, these two ER tethering regulators may act together (Sassano et al., 2023). There is currently no known association of E-Syt1 or SYNJ2BP with rare diseases.

It is expected that interfering with any of these *bona fide* MERC tethers directly affects key MERC readouts, as detected by decreased Ca^2+^ transfer, disrupted mitochondrial lipid content and altered OXPHOS activity. Where known, we will discuss how disease results from mutations in these proteins, as well as their function during homeostatic and stressed situations, where applicable (Figure 1). MERC-associated diseases are indicated in bold.

### VAPB and PTPIP51

One of the best characterized MERC tethers is formed between VAPB, an ER-localized tail-anchored protein with an N-terminal major sperm protein (MSP) domain (Dudas et al., 2021), and mitochondrial PTPIP51 (De Vos et al., 2012). Recent findings indicate PTPIP51 contains a tetratricopeptide repeat domain that mediates PA transport and controls mitochondrial CL content, which could singlehandedly explain the function of this complex for MERCs (Yeo et al., 2021). In addition to MERC tethering, VAPB also forms ER-based interorganellar protein complexes with Golgi, plasma membrane, endosome, and peroxisome proteins (Borgese et al., 2021). The VAPB-PTPIP51 heterodimeric interaction increases upon activation of GSK3β (Stoica et al., 2014). At MERCs, VAPB recognizes two motifs characterized by two phenylalanines (FF) in an acidic tract (FFAT) within PTPIP51, which then leads to the interaction of VAPB with a disordered domain within PTPIP51, thus controlling the tethering and lipid transport functions of this complex (Di Mattia et al., 2020; Yeo et al., 2021). Expression of deletion mutants of one FFAT motif within PTPIP51 also reduces the ER Ca^2+^ signaling towards mitochondria in half in SH-SY5Y cells, compared to wild-type cells (De Vos et al., 2012; Morotz et al., 2022). The VAPB-PTPIP51 tether pair is present at neuronal synapses, where it controls their activity (Gomez-Suaga et al., 2019). MERC-tethering by VAPB-PTPIP51 increases upon synaptic activity and this promotes dendritic spine number, suggesting that neuronal firing correlates with increased MERC tethering and lipid transfer through VAPB-PTPIP51 complexes (Gomez-Suaga et al., 2019).

Different from the *PTPIP51/RMDN3* gene for which there are currently no known disease-associated mutations, *VAPB* mutation is an important and frequent cause of amyotrophic lateral sclerosis (ALS), recently reviewed by us (Chen et al., 2021), leading to its alternate designation as *ALS8* (MIM#608627; Nishimura et al., 2004). However, in addition to these relatively frequent ALS-related mutations, there are about 200 cases of VAPB-based genetic disease, characterized by spinal muscular atrophy (Kosac et al., 2013). In this disease, patients suffer from progressive proximal weakness, cramps, and a lack of reflexes. These defects are likely derived from abnormal release of synaptic vesicles and aberrant endosomal trafficking. There appears to be some unclear level of overlap between ALS and this pathology, termed **Finkel-type spinal muscular atrophy (MIM#182980)**, since it can be based on the ALS-causing P56S mutation (Nishimura et al., 2004). *VAPB* mutation may largely compromise neurite elongation, presumably due to a trafficking defect based on disrupted vesicular trafficking and phosphoinositide balance, thus implicating functions of VAPB in the ER interaction with organelles other than mitochondria (Genevini et al., 2019). Therefore, mutation of *VAPB* can cause rare diseases that partially overlap with ALS, as well as with its function as a MERC tether.

### Mfn2

Amongst the more frequent MERC pathologies are mutations in the Mfn2 tether, which cause **Charcot-Marie-Tooth (CMT) disease type 2A (MIM#609260)** (Zuchner et al., 2004). This disease is a peripheral neuropathy that disrupts neuronal function in the limbs (McCray and Scherer, 2021). While a Mfn2 subpopulation localizes to the ER, especially as shorter, splicing-based variants that increase upon ER stress, to boost MERC tethering (Naon et al., 2023), its main function is based on an OMM GTPase activity that promotes mitochondria fusion (Filadi et al., 2018b). The role of impaired mitochondrial dynamics based on Mfn2 mutations has been reviewed recently (Zaman and Shutt, 2022). The *MFN2*-based type of CMT accounts for 4–7% of genetically associated CMT with an overall incidence of 1 in 2500 (Azzedine et al., 2012; Murphy et al., 2012) and the currently more than 100 known mutations are most frequently autosomal dominant, but rarely autosomal recessive with adult onset (Hikiami et al., 2018; Zaman and Shutt, 2022). CMT2A is a peripheral neuropathy that leads to distal weakness, atrophy, sensory loss, and foot deformations (Pipis et al., 2020). The majority of known mutations are found within or close to the GTPase domain, which mediates mitochondrial fusion but also controls tethering (Filadi et al., 2018b). While the mitochondria phenotype derived from CMT2A mutations shows no clear hallmark of changes of membrane dynamics *in vivo* (El Fissi et al., 2018; Ueda and Ishihara, 2018), the *MFN2* R94Q mutation associated with late-onset CMT2A shows decreased MERCs, ER stress and altered cytosolic Ca^2+^ homeostasis, associated with neurite degeneration (Bernard-Marissal et al., 2018). This could indicate that MERC defects contribute to CMT2A. This possibility was investigated in a recent report (based on *MFN2* R364W, M376V, and W740S mutations) (Larrea et al., 2019). This study found that while phosphatidylcholine to PE conversion was not changed, cholesterol ester incorporation into lipid droplets was higher in patient cells. In parallel, a small increase in respiration was found, while direct ER-mitochondria Ca^2+^ transfer was not measured (Larrea et al., 2019). From these findings we surmise that the control of mitochondrial respiration, a key function of MERCs, is not unequivocally associated with *MFN2* mutation. Consistent with this caveat, earlier studies had shown that T105M, I213T, and V273G mutations did not affect OXPHOS (Amiott et al., 2008). Together, this indicates that CMT2A is associated with modestly changed MERC functions, and point to an overall high degree of variability of the disease characteristics with regard to MERC involvement (Zaman and Shutt, 2022). Pharmacological approaches to treat CMT2A include Mfn2 agonists such as MiM111, which can improve mitochondrial fitness in a mouse preclinical T105M model (Franco et al., 2020). Due to the lack of a known connection between the T105M mutation with MERCs, the extent of rescue of any MERC dysfunction was not investigated in this study. Moreover, we currently do not know whether the recently discovered splice variants mediating MERC tethering (Naon et al., 2023) are affected in a specific manner in CMT2A.

### IP_3_R-VDAC-GRP75 (HSPA9) Tether

A ternary tethering complex forms between the IP_3_R1 isoform with the voltage-dependent anion channel 1 (VDAC1) and the outer mitochondrial membrane chaperone GRP75 (Szabadkai et al., 2006). It is currently not clear whether IP_3_R2 and IP_3_R3 can also do this, however, these forms have been reported to transmit Ca^2+^ signals from the ER to mitochondria more effectively (Mendes et al., 2005; Bartok et al., 2019). In contrast to VDAC, several GRP75 and IP_3_R-based rare genetic diseases have been reported to date.

IP_3_Rs are expressed from three gene loci, *ITRP1* (IP_3_R1), *ITRP2* (IP_3_R2), and *ITRP3* (IP_3_R3). Although these paralogs are overall similar in amino-acid sequences with each other, they exhibit distinct characteristics such as interaction partners and expression patterns. For example, IP_3_R1 dominates over the other forms in cerebellum and brain (Wojcikiewicz, 1995), while IP_3_R2 dominates in the liver and heart (Ivanova et al., 2014).

A gain-of-function mutation in the suppressor domain of the IP_3_R1 (R36C) causes **spinocerebellar ataxia** (MIM#117360). This neurological disorder is characterized by early motor delay, poor coordination, gait ataxia, and dysarthria (Casey et al., 2017). The N-terminal suppressor domain normally limits ER Ca^2+^ release in the presence of IP_3_ (Szlufcik et al., 2006). Additional mutations are found in the ligand-binding domain (Terry et al., 2018). Both types of mutants typically exhibit higher IP_3_ binding affinity and Ca^2+^ release to the cytoplasm upon stimulation, increasing both the peak amplitude and the amount of Ca^2+^ release.

In contrast to these gain-of-function mutations, dominant-negative IP_3_R1 mutations cause **Gillespie syndrome (MIM#206700)**, characterized by bilateral iris hypoplasia, nonprogressive ataxia, and progressive cerebellar atrophy (Gerber et al., 2016; McEntagart et al., 2016). When those IP_3_R1 mutants are expressed in the presence of the wild-type IP_3_R1, the Ca^2+^ channel function of IP_3_R is reduced. Both gain-of-function and dominant-negative mutants indicate that properly controlled Ca^2+^ signaling from the ER is critically important for cerebellar and brain development.

Like *ITRP1*, *ITRP3* mutations are known to cause neuronal and developmental disorders. Dominant mutations in *ITPR3* (e.g., V615 M) cause **CMT1J (MIM#620111)**, resulting in altered Ca^2+^ signaling upon various stimuli compared to control fibroblasts (Ronkko et al., 2020). Unlike its paralogs, IP_3_R3 is preferentially expressed in Schwann cells that protect neurons (Toews et al., 2007). Individual mutant IP_3_R paralogs can assemble into homo- and hetero-tetramers to form semi-functional Ca^2+^ channels (Nucifora et al., 1996), thus explaining these rather uniform effects on Ca^2+^ signaling in the presence of wild-type copies of IP_3_R proteins.

GRP75, also known under the names of mtHsp70, HSPA9, and mortalin, is a chaperone that mainly localizes to mitochondria (Havalova et al., 2021), where it protects from ROS and controls mitochondrial biogenesis, including protein translocation into mitochondria, and the synthesis of Fe–S cluster proteins (Flachbartova and Kovacech, 2013). GRP75 localizes to multiple intracellular compartments. While the main localization of GRP75 is on mitochondria, ∼30% of its amount is found in other compartments (Esfahanian et al., 2023), including endosomes, the cytosol, the ER and the MAM, where it forms the IP_3_R1-GRP75-VDAC tethering complex (Szabadkai et al., 2006). Several genetic diseases are caused by mutations in the *GRP75* gene. One type causes the **EVEN-PLUS syndrome**, causing severe microtia, nasal hypoplasia, and other malformations in addition to ocular, dental, auricular, and skeletal defects, but not mental retardation (Royer-Bertrand et al., 2015). The name of this disease stems from defects of the **E**piphyses, **V**ertebrae, **E**ars, and **N**ose, **PLUS** other issues. Most mutations affect the structure of the nucleotide-binding domain, presumably affecting the GRP75 ATPase activity (Moseng et al., 2019). Additional mutations can also compromise the amounts of mRNA (Younger et al., 2020). Interestingly, this pathology resembles CODAS syndrome (MIM#600373; cerebral, ocular, dental, auricular, and skeletal) caused by mutations in the *LONP1* gene, which codes for a mitochondrial chaperone and protease (Dikoglu et al., 2015; Strauss et al., 2015). This protease has been detected on MERCs (Horner et al., 2015) and its transcription specifically increases upon ER stress induction (Hori et al., 2002). Once induced, LonP1 protects mitochondrial homeostasis by degrading oxidized proteins (Bota and Davies, 2002), confirming that ER stress-triggered transcriptional responses aim to maintain mitochondrial functionality during its early stages, as recently described by us (Bassot et al., 2023). Additional mutations of GRP75 have been described to cause congenital sideroblastic anemia (MIM#182170; Schmitz-Abe et al., 2015). Patients of this type of genetic disease exhibit specific defects in mitochondrial heme synthesis, and Fe–S cluster protein biogenesis. Despite these differences, this rare disease also highlights the predominant function of GRP75 for mitochondrial homeostasis, but it is unclear whether this is based on its tethering or chaperoning function. Nevertheless, IP_3_R and GRP75 mutations both affect the CNS/PNS functioning to a significant extent.

### BAP31 (BCAP31)

In cells depleted of the MERC regulatory protein PACS-2, the ER protein BAP31 results as cleaved, which no longer allows it to interact with mitochondrial proteins such as Fis1 or Tom40 (Simmen et al., 2005). Mutation of *BCAP31* is observed in a rare X-linked recessive disorder termed **deafness, dystonia, and central hypomyelination (DDCH)** syndrome (MIM#300475) that results in loss of function (Cacciagli et al., 2013). DDCH is also known under the name of Schimke X-linked intellectual disability (XILD) (Louie et al., 2020). Consistent with its role as a MERC tether, BAP31 depletion can trigger ER stress (Xu et al., 2019), and interferes with MERC Ca^2+^ signaling (Iwasawa et al., 2011), as well as lipid production and transfer (Wei et al., 2023). BAP31 is also required for normal mitochondrial OXPHOS (Namba, 2019; Shimizu et al., 2020). BAP31 mutation results in the alteration of the ER structure that becomes swollen and reduces secretory protein production, thus potentially explaining hypomyelination observed in DDCH (Cacciagli et al., 2013). BAP31 also determines the expression of valosin-containing protein (VCP) (Jia et al., 2018). This ubiquitin-specific chaperone controls proteasomal protein degradation at the ER (Wojcik et al., 2006), and mitochondria (Xu et al., 2011). VCP executes this function through interaction with the E3 ubiquitin ligase Gp78/AMFR (Zhong et al., 2004) that controls Mfn2 levels (Wang et al., 2015). Therefore, BAP31 determines MERC function through multiple mechanisms and its mutation affects the CNS.

### PDZD8/VPS13

The mammalian SMP domain-containing protein PDZD8 was first described as an ER-mitochondria tether, mediating efficient Ca^2+^ flux from the ER to mitochondria in neuronal cells (Hirabayashi et al., 2017). The presence of an SMP domain suggests that PDZD8 may use its localization to ER membrane contact sites to mediate exchange of lipid molecules at these locations (Jeyasimman and Saheki, 2020). This function is particularly evident in yeast, where in addition to the PDZD8 paralog Mmm1 (Hirabayashi et al., 2017; Wideman et al., 2018), several SMP domain-containing proteins (Mdm10, Mdm12, and Mdm34) form the ERMES complex, which mediates lipid exchange between the ER and mitochondria (Kornmann et al., 2009). Premature stop codons in the *PDZD8* gene create a shortened version of this tether that is associated with intellectual disability designated as “**intellectual developmental disorder with autism and dysmorphic facies (IDDADF)” (MIM#620021)** (Al-Amri et al., 2022). In contrast, PDZD8 promotes age-associated decline in locomotor activity in a *Drosophila melanogaster* model (Hewitt et al., 2022), suggesting that its activity must be closely titrated during aging to allow for normal neuronal function. However, PDZD8 is not just controlling MERCs but also fulfills similar functions on membrane contacts with endosomes (Guillen-Samander et al., 2019; Shirane et al., 2020), thus making a clear association of IDDADF with MERCs unclear. There is potential pathologic crosstalk of PDZD8 with VPS13A, whose mutations cause hyperkinetic movements and cognitive abnormalities in **chorea acanthocytosis (MIM#200150)** (Yeshaw et al., 2019). VPS13A mediates mitochondria–endosome contacts, while the closely related VPS13D interacts with Miro-1 and thus promotes the formation of a triple ER-mitochondria–peroxisome contact site (Guillen-Samander et al., 2021). On ER contact sites, VPS13D spans the interorganellar cleft and is able to transfer large amounts of lipids toward mitochondria, as is typical for VPS13 family proteins (Melia and Reinisch, 2022). Mutations within VPS13D lead to a rare disease with chorea, dystonia, spastic ataxia, and spastic paraplegia (Koh et al., 2020), as well as **spinocerebellar ataxia (SCA) recessive type 4 (SCAR4)** (Seong et al., 2018). In both cases, the disease onset may occur during childhood or adult age (Chang et al., 2023). As expected from a MERC disorder that also compromises mitochondria, SCAR4 patient cells show abnormal mitochondrial positioning, membrane dynamics, and bioenergetics (Koh et al., 2020).

## Rare Diseases Based on MERC-Mediated Mitochondrial Dynamics

The role of MERCs for mitochondrial dynamics is evident in the case of mitochondrial fission, where ER-associated Drp1 becomes enriched on MERCs and forms a constricting ring with the associated ER membranes around a mitochondrion (Kalia et al., 2018). This mechanism requires ER-associated actin polymerization (Korobova et al., 2013) and mitochondrial CL (Stepanyants et al., 2015), thus highlighting the high significance of MERC formation when disease is associated with mitochondrial hyperfusion from disrupted fission.

### Drp1

The ER GTPase Drp1 is mutated in a lethal encelopathy of early childhood (Waterham et al., 2007), a spectrum characterized by epilepsy, and microcephaly (Navaratnarajah et al., 2021). The disease resulting from *DRP1* mutations is referred to as **encephalopathy due to defective mitochondrial and peroxisomal fission 1 (EMPF1, MIM#614388)** (Assia Batzir et al., 2019). EMPF1 patient cells show elongated mitochondrial morphology, increased mitochondrial proton leak and upregulation of glycolysis. However, this mitochondrial phenotype is not clean due to the association of Drp1 with peroxisome fission and also comprises severely elongated peroxisomes (Robertson et al., 2023). While it is not known whether patient cells show MERC defects, Drp1 crosstalks with oxidative stress and could thus be under the influence of MERC ROS signaling. Accordingly, its activating phosphorylation state is promoted by nitrosylation (Cho et al., 2009) and sulfenylation (Kim et al., 2018), potentially due to activation of protein kinase A (PKA) by oxidation (Brennan et al., 2006). Conversely, Drp1 silencing reduces ROS production (Roth et al., 2014), while its increased expression increases ROS (Xu et al., 2022). Moreover, lipid homeostasis at MERCs influences Drp1 activity. This is based on the requirement of mitochondrial fission for efficient lipid usage for mitochondrial fatty acid oxidation (Song et al., 2021). Consistent with this, Drp1-mediated fission is activated upon cellular loading of a variety of fatty acids (Zezina et al., 2018). These findings highlight the high level of connectivity between MERC functions in this case but also in general.

### Mff

The assembly of mitochondrial receptors is critical for Drp1 oligomerization. The mitochondrial fission factor (Mff) is the primary mitochondrial receptor for Drp1 (Loson et al., 2013). Its targeting to the ER portion of MERCs greatly increases mitochondrial fission (Ji et al., 2017) Neuronal defects result, for instance, upon mutation of the MFF gene in the **Leigh-like basal ganglia disease-optic atrophy-peripheral neuropathy syndrome,** also referred to as **encephalopathy due to defective mitochondrial and peroxisomal fission 2 (EMPF2, MIM#617086)**, due to defective mitochondrial and peroxisomal fission (Shamseldin et al., 2012). This disease starts at an early age with seizures, a developmental delay and acquired microcephaly, while not affecting skeletal muscle. At a later stage, spasticity and optic and peripheral neuropathy develop (Koch et al., 2016). The observed defects appear largely tied to mitochondrial defects, associated with high lactate levels within the cerebrospinal fluid, and do not show abnormal peroxisome activity (Nasca et al., 2018).

## Rare Diseases from Mutated MERC Regulatory or Accessory Proteins

Several proteins that modulate MERC signaling through changes in activity of ER-mitochondria lipid flux, as well as ER Ca^2+^ pumping and release have been described over the past decade. They include calnexin (Lynes et al., 2012), TMX1 (Raturi et al., 2016), ERdj5 (Ushioda et al., 2016), and GPx8 (Yoboue et al., 2017). Their activity and localization is controlled by cytosolic factors such as PACS-2 (Myhill et al., 2008), as well as by the ER membrane protein complex (EMC) (Christianson et al., 2011). Some of these regulatory proteins and their interactors are listed below, where we discuss their association with MERC-connected rare diseases.

### PACS-2

PACS-2, the first identified regulator of MERC tethering, maintains MERC protein complexes by preventing caspase-8-mediated cleavage of BAP31, thus preserving the MERC structure (Simmen et al., 2005). PACS-2 is phosphorylated during homeostatic conditions (Betz et al., 2013) and promotes MERC lipid synthesis as well as Ca^2+^ signaling during apoptosis (Simmen et al., 2005; Aslan et al., 2009). PACS-2 also controls the MERC localization of important functional and regulatory proteins, including ACSL4 and calnexin (Simmen et al., 2005). Like many proteins that are subject of this review, PACS-2 is multifunctional (Thomas et al., 2017). Its anabolic, pro-survival, and apoptotic functions are controlled by Akt phosphorylation (Aslan et al., 2009). Additionally, PACS-2 can migrate into the nucleus, where it prevents DNA damage through p53 and p21 and controls ATM trafficking toward the cytosol (Barroso-Gonzalez et al., 2016). In the absence of PACS-2, the histone deacetylase sirtuin-1 (SIRT1) is hyperactive, which normally maintains p53-mediated transcription (Atkins et al., 2014). Importantly, however, some of the nuclear functions of PACS-2 may indirectly depend on its MERC functions: disrupted ER-mitochondria Ca^2+^ flux, followed by mitochondrial fragmentation, as observed upon PACS-2 knockdown, activates SIRT1 (Lovy et al., 2020). Moreover, increased ROS content upon PACS-2 knockdown could migrate into the nucleus and promote DNA damage (Srinivas et al., 2019).

PACS-2 is mutated in PACS-2 syndrome, a subtype of early infantile developmental and epileptic encephalopathy (EIDEE) (Zuberi et al., 2022), referred to as **developmental and epileptic encephalopathy-66 (DEE66**—MIM#618067). Amongst the monogenic DEE subtypes, several include mutations within the genes encoding ion channels and synaptic vesicle trafficking factors (Syrbe, 2022), indicating that *PACS-2*-based DEE66 might depend on the trafficking functions of this cytosolic protein, in addition to its functions at MERCs. The most frequently observed *PACS-2* mutation is E209K, characterized by seizures, behavioral abnormalities, developmental delay with hypotonia, facial dysmorphism, and ophthalmologic defects (Olson et al., 2018). Additional mutations have been identified to result in E211K (Dentici et al., 2019). Patients start to suffer from epilepsy within a few days after birth but these symptoms can be addressed with vitamin B6 (Chou et al., 2023). Consistent with the hypothesis that *PACS-2* mutation results in dysfunctional MERCs, vitamin B6 protects iron–sulfur cluster proteins found within complex I and II of the mitochondrial OXPHOS electron transport chain, thus rescuing potential MERC signaling towards OXPHOS (Braymer and Lill, 2017). Additional mutations have been identified to result in E211K (Dentici et al., 2019).

PACS-2 depletion causes ER stress, reduces PSS1 at MERCs and disrupts Ca^2+^ signaling (Simmen et al., 2005). This is also observed in DEE66 mutant cells (Thi My Nhung et al., 2023). PACS-2 also controls the pro-apoptotic trafficking of the Bcl2 family protein Bim onto mitochondria and lysosomes (Simmen et al., 2005; Werneburg et al., 2012) and is also found on endosomes, where it can influence major histocompatibility complex class I (MHC-I) downregulation (Atkins et al., 2014). Moreover, PACS-2 also promotes MERC-localized mitophagy in vascular smooth muscle cells (Moulis et al., 2019). Yet, its role for MERCs is likely a main function. As such, PACS-2 interacts with coatomer and retrieves MERC-regulatory proteins such as the transient receptor potential polycystin-2 (TRPP2) and calnexin from the Golgi complex to the ER, where they then get enriched on MERCs (Kottgen et al., 2005; Myhill et al., 2008). Both PACS-2 cargo proteins promote ER-mitochondria Ca^2+^ communication: TRPP2 likely controls this signaling mechanism through a control of MERC IP_3_R activity and downregulates Mfn2 (Kuo et al., 2019), while calnexin promotes oxidation of MAM Ca^2+^ handling proteins such as SERCA and IP_3_Rs, thus activating their signaling functions (Gutierrez et al., 2020).

In the *PACS-2*-dependent disease scenario, mutant PACS-2 protein increases apoptosis susceptibility (Zang et al., 2022). While mutant PACS-2 could potentially fail to stabilize ion channels such as TRPP2 (Kottgen et al., 2005), another mechanistic basis of this syndrome could be an increased half-life of mutant PACS-2, leading to its abnormal accumulation that would then abnormally promote MERC formation (Zang et al., 2022). As hypothesized recently, mutant PACS-2 could also change ion channel activity at MERCs or elsewhere (Chou et al., 2023) or modulate MERC-derived autophagy (Hamasaki et al., 2013). This latter function also connects PACS-2 to the prevention of cardiac injury, especially under hypoxic conditions at high altitude, where it helps maintain normal cardiomyocyte metabolism (Yang et al., 2023).

### EMC

The EMC is a multiprotein transmembrane complex that promotes the insertion of transmembrane proteins (Guna et al., 2017). This function determines largely the topology of multipass transmembrane proteins (Shurtleff et al., 2018). An important EMC substrate is rhodopsin that is compromised upon EMC3 interference (Taylor et al., 2005). Interestingly, EMC interacts with calnexin (Christianson et al., 2011) that can show high levels of MERC enrichment (Lynes et al., 2013). Consistent with functions of EMC for MERCs, this protein complex also determines MERC formation itself and its knockout compromises PS transfer from the ER to mitochondria at MERCs (Lahiri et al., 2014). At the moment, it is unclear whether this function stems from a direct role of EMC for MERCs, or, more likely, from the insertion of MERC tethers at the level of the ER. Accordingly, genetic diseases associated with *EMC* mutation are most closely associated with membrane protein insertion, such as *EMC3* mutation that is expected to affect vision (Cao et al., 2021). Nevertheless, mutations within the *EMC1* and *EMC10* genes lead to overlapping disease spectrums, highly reminiscent of MERC disorders (Chung et al., 2022): while EMC1 mutation causes **cerebellar atrophy, visual impairment and psychomotor retardation (CAVIPMR) (MIM#616875)** (Harel et al., 2016; Geetha et al., 2018), **EMC10 mutation causes neurodevelopmental disorder with dysmorphic facies and variable seizures (NEDDFAS) (MIM#619264)** (Umair et al., 2020; Shao et al., 2021).

### SEPN1

An interesting pair of ER oxidoreductases is formed by ER oxidoreductin 1 (ERO1) and SELENON, also known as Selenoprotein N1 (SEPN1). ERO1 can oxidize SERCA, which impairs ER Ca^2+^ filling (Marino et al., 2015), necessary for normal MERC function (Gutierrez et al., 2020). The oxidizing enzyme ERO1 has long been recognized as an important component of MERCs whose enrichment is oxygen and redox-dependent (Gilady et al., 2010). ERO1 oxidizes proteins involved in tethering during the onset of ER stress (Bassot et al., 2023). In addition to tethers, ERO1 also targets IP_3_Rs and results in their activation through oxidation (Li et al., 2009). Consequently, ERO1 activates mitochondria, especially under ER stress (Bassot et al., 2023).

In contrast, SELENON/SEPN1 is an ER antioxidant protein (Arbogast et al., 2009), whose mutant variants are found in **SEPN1-related myopathy (SEPN1-RM, MIM#602771)** (Villar-Quiles et al., 2020). Different from MERC tethering defects that predominantly lead to CNS developmental and functional defects, this disease is characterized by rigid spine muscular dystrophy, and multiminicores, congenital fiber type disproportion, and desmin-related myopathy (Moghadaszadeh et al., 1998, 2001; Ferreiro et al., 2002). On a cellular level, SEPN1-RM is characterized by a depletion of mitochondria in terms of number but also function, since the cellular ATP content is less, based on reduced MERC Ca^2+^ signaling (Filipe et al., 2021). In contrast to ERO1, SEPN1 appears specific for SERCA and does not affect IP_3_Rs, thus potentially explaining the similar readout in terms of mitochondrial bioenergetics despite its antioxidant function versus oxidizing ERO1 (Pozzer et al., 2019).

### GDAP1 (CMT4A)

**Charcot-Marie-Tooth (CMT) disease 4A (MIM#214400)** is an autosomal-recessive polyneuropathy caused by mutations in ganglioside-induced differentiation-associated protein 1 (GDAP1) (Bird, 1993). The transmembrane GDAP1 protein promotes mitochondrial fission from its OMM localization, but it does not influence mitochondrial fusion (Niemann et al., 2005). It exposes two glutathione S-transferase domains to the cytosol, but there is still a debate about whether it maintains an ability to bind glutathione (Googins et al., 2020). Its presence typically increases the amount of GSH (Noack et al., 2012), while its knockout increases GSSG in a mouse model (Niemann et al., 2014). However, the catalytic cysteines within the GST domains are not conserved, suggesting functions not related to an enzymatic activity might be responsible for these observations (Wolf et al., 2020). An alternative function responsible a function of GDAP1 in mitochondrial fission could be based on its interaction with Cofilin-1, the F-actin interacting protein. GDAP knockdown reduces F-actin close to mitochondria, increases membrane distance at MERCs and reduces the mitochondrial Ca^2+^ level as well as the activity of pyruvate dehydrogenase (Wolf et al., 2022). This could be based on altered mitochondrial distribution and disrupted ER Ca^2+^ flux dynamics at MERCs (Bird, 1993). Such a disruption of MERCs is a known cause for redox imbalance, as shown in numerous examples, thus suggesting that it predominantly acts as a MERC distance regulator.

### PSS1/PSS2/PISD

As mentioned in the introduction, a main function of MERCs is the transformation of PS into PE, the original function discovered by Jean Vance (Vance, 1990, 1991). Both PSS1 and PSS2 localize to MERCs (Stone and Vance, 2000). A *D. melanogaster* knockout results in altered mitochondrial morphology and increased ROS production (Park et al., 2021), as is typical when the MERC structure undergoes functional changes, such as the activation of ER-mitochondria Ca^2+^ flux (Booth et al., 2016). Human patients with a *PTDSS1* mutation suffer from **Lenz-Majewski syndrome (MIM#151050)**, a rare disease characterized by multiple developmental abnormalities including intellectual disabilities, progressive skeletal sclerosis, cutis laxa, dental enamel dysplasia, and abnormal development in skull and fingers (Sohn and Balla, 2016).

Likewise, PISD activity that converts PS to PE on the IMM in proximity to MERCs is required for mitochondrial OXPHOS at complex I and IV, as well as normal mitochondrial membrane potential (Tasseva et al., 2013). Unlike the viable PSS1 and PSS2 knockout animals (Bergo et al., 2002; Arikketh et al., 2008), the activity of PISD is essential, since it determines embryonic development and mitochondrial integrity (Steenbergen et al., 2005). *PISD* mutation-associated pathologies have been classified as mitochondrial diseases with skeletal and central nervous system (CNS) dysfunction, characterized by hypomyelination, ataxia, and intellectual disability (Zhao et al., 2019). This rare disease is now referred to as **Liberfarb syndrome (MIM#618889)** (Peter et al., 2019). Overall, both Lenz-Majewski and Liberfarb syndromes fit nicely into the previously discussed MERC pathologies.

### ACSL4 (FACL4)

Acyl CoA synthetase 4 (ACSL4) is a reliable marker of MERCs (Lewin et al., 2002). This enzyme promotes the production of PUFAs through the incorporation of arachidonic acid into phospholipids (Kang et al., 1997; Golej et al., 2011). Mutations in the *ACSL4* gene are found in patients with **X-linked intellectual developmental disorder 63 (XLID63, MIM#300387)** (Meloni et al., 2002). At the moment, it is unclear whether this disease phenotype stems from roles of ACSL4 at and for MERCs or from roles in ferroptosis (Doll et al., 2017).

## Concluding Remarks and Potential Therapeutic Avenues

**Figure 2.**
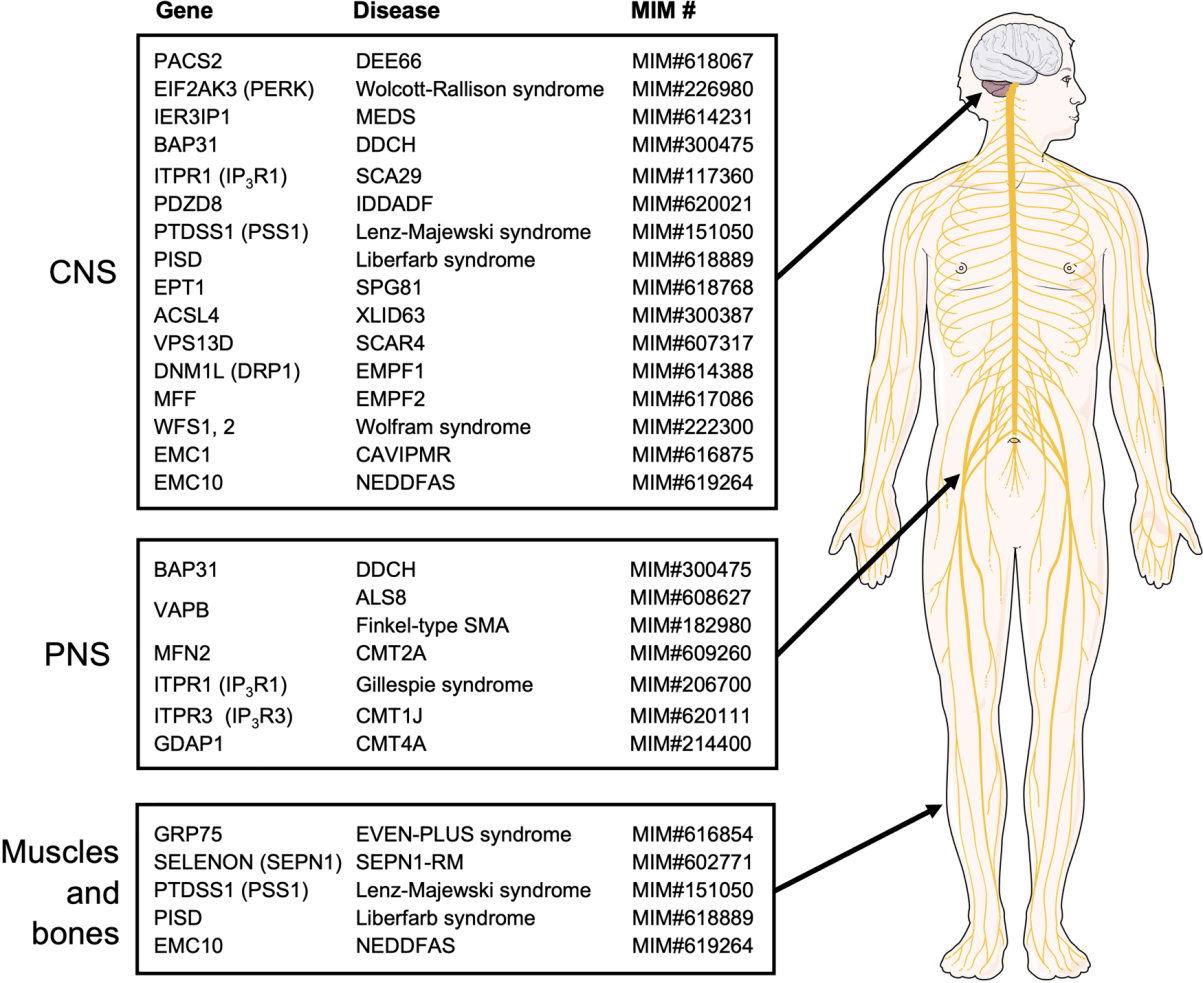
Rare diseases caused by mutations in the MERC-related genes. The MERC-related genes whose mutations cause rare diseases are summarized. They are categorized by the systems mostly affected by their respective diseases. The MIM numbers of the genetic diseases given by OMIM (https://www.omim.org/) are also shown. Please note that additional systems may be affected by a disease. The illustration is adapted from Servier Medical Art (https://smart.servier.com/).

*Note*. MERC: mitochondria-endoplasmic reticulum contact; MIM: Mendelian Inheritance in Man; CNS: central nervous system; PNS: peripheral nervous system; DEE: developmental and epileptic encephalopathy; MEDS: microcephaly, epilepsy, and diabetes syndrome; DDCH: deafness, dystonia, and cerebral hypomyelination; SCA: spinocerebellar ataxia; IDDADF: intellectual developmental disorder with autism and dysmorphic facies; SPG: spastic paraplegia; XLID: X-linked intellectual developmental disorder; EMPF: encephalopathy due to defective mitochondrial and peroxisomal fission; CAVIPMR: cerebellar atrophy, visual impairment and psychomotor retardation; NEDDFAS: neurodevelopmental disorder with dysmorphic facies and variable seizures; ALS: amyotrophic lateral sclerosis; SMA: spinal muscular atrophy; CMT: Charcot-Marie-Tooth disease; EVEN-PLUS: epiphyses, vertebrae, ears, and nose, PLUS other issues; SEPN1-RM: Selenoprotein N1-related myopathy.

The past decade has seen increasing insight into rare diseases stemming from MERC disruption. An overall characteristic of these diseases emerges as predominantly resulting in neurological defects, with less frequent skeletal muscle deficiency (Figure 2). In many ways, PACS-2-linked DEE66 is the prototype of these pathologies with a developmental delay, intellectual disability, facial dysmorphism, and ophthalmologic defects. Additional symptoms include seizures, epilepsy, deafness, ataxia, or peripheral neuropathy. The key example of muscle defects is seen with SEPN1-RM that results in rigid spine muscular dystrophy. Of course, the insight that MERC defects lead to common characteristics in rare diseases opens up the possibility to find common or similar treatment avenues. At the moment, however, it is difficult to foresee whether intervention at the level of lipid, Ca^2+^, or ROS functions of MERCs will be most successful. Therefore, current treatment options include vitamin B6 in the case of DEE6 (Chou et al., 2023). This is based on the role of this vitamin for OXPHOS through its control of iron–sulfur and NAD+ biosynthesis (Ciapaite et al., 2023). Another example is Mfn2 agonists such as MiM111 that aim to preserve mitochondrial bioenergetics (Franco et al., 2020). Similarly, Drp1 inhibition with the cell-permeable quinazolinone Mdivi-1 could address some of the diseases described here (Xie et al., 2016).

MERC dysfunction could respond to increased or decreased SERCA activity, as well as altered IP_3_R Ca^2+^ release. While experimental cancer therapeutics make use of this idea through inhibition of SERCA and presumably MERCs, the activation of SERCA could also be promising (Tadini-Buoninsegni et al., 2018). This latter drug group, for instance the allosteric activator CDN1163 (Kang et al., 2016), could be of particular interest in the case of SEPN1-RM. Another approach in this context is the use of IP_3_R-modulating agonists, like adenophostins (Gambardella et al., 2021), and antagonists, like the macrocyclic bis-1-oxaquinolizidine xestospongin, which induce relaxation of blood vessels *in vivo* (Nakagawa et al., 1984). However, IP_3_R-modulatory compounds currently seem to suffer from a lack of specificity. Future research will have to determine whether compounds or proteins based on the endogenous IP_3_R antagonist IRBIT (IP_3_R-binding protein released with IP_3_R) could be more specific (Ando et al., 2003, 2006).

Additionally, ROS intervention appears an obvious approach, due to the wide availability of pro- and antioxidants, which, however, would have to be targeted to the mitochondria of the tissue affected most by any rare disease (Jiang et al., 2020). Such approaches are considered, for instance, in the case of SEPN1-RM (Arbogast et al., 2009). However, ROS may increase or decrease upon MERC interference, therefore lipid and Ca^2+^ flux changes currently seem more straightforward. Given that PE defects are most closely associated with MERC and mitochondrial dysfunction, *in vitro* supplementation with ethanolamine and lyso-PE appears a promising approach (St Germain et al., 2022). However, in the case of *PISD*-mutant *in vitro* studies, this approach required the additional modulation of ROS (Girisha et al., 2019) and lyso-PE supplementation can cause liver disease (Yamamoto et al., 2022). An indirect approach could be the use of peroxisome proliferators that increase, ER, mitochondria, and peroxisome function in the liver to modulate PE metabolism (Mizuguchi et al., 1999).

Not all MERC proteins fit into our proposed common MERC-disease pattern. For instance, the PERK-connected Wolcott Rallison syndrome has a predominant diabetic consequence. At this point, we hypothesize that such discrepancies stem from the predominant function of the respective protein for functions other than MERC formation or regulation, in the case of PERK as a regulator of the UPR. Similarly, problems with vision upon EMC protein mutation are likely connected to the role of EMC for rhodopsin, while the weakness, cramps, and a lack of reflexes in the case of VAPB could derive from the functions of this tether in ER MCS with organelles other than mitochondria. The further use of animal models, further insight about the global functions of MERC proteins and the advent of new compounds will provide the necessary insight required for testing any of these approaches based on MERC ROS, lipid, and Ca^2+^ signaling.
