# Erratum to “Not so Rare: Diseases based on Mutant Proteins Controlling Endoplasmic Reticulum-Mitochondria Contact (MERC) Tethering”

**DOI:** 10.1177/25152564241276081

**Published:** 2024-09-24

Makio T, Simmen T (2024). Not so rare: Diseases based on mutant proteins controlling Endoplasmic Reticulum-Mitochondria Contact (MERC) tethering. *Contact* 7. doi:10.1177/25152564241261228

In this article, the article title has been corrected from “The Discovery of Mitochondria-Endoplasmic Reticulum Contact Sites (MERCs) as Mitochondria-Associated Membranes (MAMs)” to “Not so Rare: Diseases based on Mutant Proteins Controlling Endoplasmic Reticulum-Mitochondria Contact (MERC) Tethering”.

